# Clodronate Reduces ATP-Containing Microvesicle Releasing Induced by Nociceptive Stimuli in Human Keratinocytes

**DOI:** 10.3390/ijms25158435

**Published:** 2024-08-02

**Authors:** Filippo Renò, Marco De Andrea, Stefano Raviola, Mario Migliario, Marco Invernizzi

**Affiliations:** 1 Department of Health Sciences, University of Milan, Via A. di Rudini 8, 20142 Milan, Italy; 2 Center for Translational Research on Autoimmune and Allergic Disease (CAAD), University of Eastern Piedmont, Corso Trieste, 15/A, 28100 Novara, Italy; marco.deandrea@unito.it (M.D.A.); stefano.raviola@uniupo.it (S.R.); 3 Department of Public Health and Pediatric Sciences, University of Turin, Via Verdi 8, 10124 Turin, Italy; 4 Department of Translational Medicine, University of Eastern Piedmont, Via Solaroli 17, 28100 Novara, Italy; mario.migliario@med.uniupo.it; 5 Department of Health Sciences, University of Eastern Piedmont, Via Solaroli 17, 28100 Novara, Italy; marco.invernizzi@uniupo.it

**Keywords:** pain, ATP, keratinocyte, microvescicles, clodronate, VNUT, capsaicin, KOH

## Abstract

Clodronate (Clod), a first-generation bisphosphonate, acts as a natural analgesic inhibiting vesicular storage of the nociception mediator ATP by vesicular nucleotide transporter (VNUT). Epidermal keratinocytes participate in cutaneous nociception, accumulating ATP within vesicles, which are released following different stimulations. Under stress conditions, keratinocytes produce microvesicles (MVs) by shedding from plasma membrane evagination. MV secretion has been identified as a novel and universal mode of intercellular communication between cells. The aim of this project was to evaluate if two nociceptive stimuli, Capsaicin and Potassium Hydroxide (KOH), could stimulate MV shedding from human keratinocytes, if these MVs could contain ATP, and if Clod could inhibit this phenomenon. In our cellular model, the HaCaT keratinocyte monolayer, both Capsaicin and KOH stimulated MV release after 3 h incubation, and the released MVs contained ATP. Moreover, Clod (5 µM) was able to reduce Caps-induced MV release and abolish the one KOH induced, while the Dansylcadaverine, an endocytosis inhibitor of Clod uptake, partially failed to block the bisphosphonate activity. Based on these new data and given the role of the activation of ATP release by keratinocytes as a vehicle for nociception and pain, the “old” bisphosphonate Clodronate could provide the pharmacological basis to develop new local analgesic drugs.

## 1. Introduction

Bisphosphonates are drugs widely used in clinics to reduce bone resorption through the induction of apoptotic phenomena in osteoclasts, thus preventing tumor-induced bone lesions [1]. On the basis of clinical evidence, a possible use of bisphosphonates as analgesics in the treatment of pain associated with bone metastases has been hypothesized [2]. Based on their chemical structure, bisphosphonates are divided into amino-bisphosphonates (N-BPs), very powerful in counteracting bone resorption but capable of causing inflammatory and necrotic effects, and bisphosphonates not containing amino groups (non-N-BPs), with less powerful antiresorptive activity, but capable of counteracting the side effects of N-BPs [3]. In particular, two non-N-BPs, etidronate and Clodronate, exert notable effects of counteracting the side effects of N-BPs by penetrating the soft tissue cells through transporter proteins of the SLC (Solute Carrier) family, the same ones used by N-BPs. The action of counteracting the effects of N-BPs by non-N-BPs would therefore take place through the competitive inhibition of SLC action [4]. As regards the analgesic action of non-N-BPs, a direct action toward neurons has been demonstrated in mice [5] for Etidronate and Clodronate that use SLC transporters (SLC20/34) and inhibit the release of pain mediators, glutamate, and ATP through an action on SLC17 [6].

The contribution of keratinocytes to cutaneous nociception has become better understood over the past decade. While intraepidermal nerve endings have traditionally been considered exclusive transducers of noxious cutaneous stimuli, it has now been demonstrated that epidermal keratinocytes can initiate nociceptive responses [7] as they can accumulate ATP [8] as neurons [9] within vesicles that are released following different stimulations, including nociceptive ones. In fact, the depolarization of the surface of keratinocytes is able to induce the release of ATP and glutamate [10], and both epidermal keratinocytes and HaCaT cells, spontaneously immortalized human keratinocytes, are capable of producing glutamate [11] and ATP [12] and releasing them following different stimuli [13,14]. Furthermore, ATP released by keratinocytes has been shown to act as a messenger for sensory neurons [14,15].

Microvesicles (MVs) are fragments released from the plasma membrane evaginations of all cell types both in basal condition and when subjected to oxidative or mechanical stress [16] during apoptosis or necrosis, and they have proven to be true vectors for the exchange of biological information between cells [17], even if they are rather heterogeneous in terms of size (0.05–0.1 µm) and protein and lipid composition. MVs have not been associated with nociception, even if an increasing number of publications describe the presence of purinergic enzymes such as CD39 and CD73 [18] as well as of ATP in MVs [19].

Given the role of the activation of keratinocytes and their release of ATP as a vehicle for nociception and pain, we evaluated if nociceptive stimuli could increase MV release in human keratinocytes, if Clodronate, a well-known non-N-BP pain inhibitor, could modulate it, and finally, if MVs could transport ATP. These data could shed light on a new Clodronate use as a local analgesic capable of modifying pain transduction [20].

## 2. Results

### 2.1. Nociceptive Stimuli Toxicity

In this study, we utilized nociceptive stimuli normally used in vivo in animal models [21,22] and adapted them to an in vitro model, i.e., human keratinocytes grown to produce a cell monolayer and whose growth was slowed down and synchronized through starvation.

**Figure 1:**
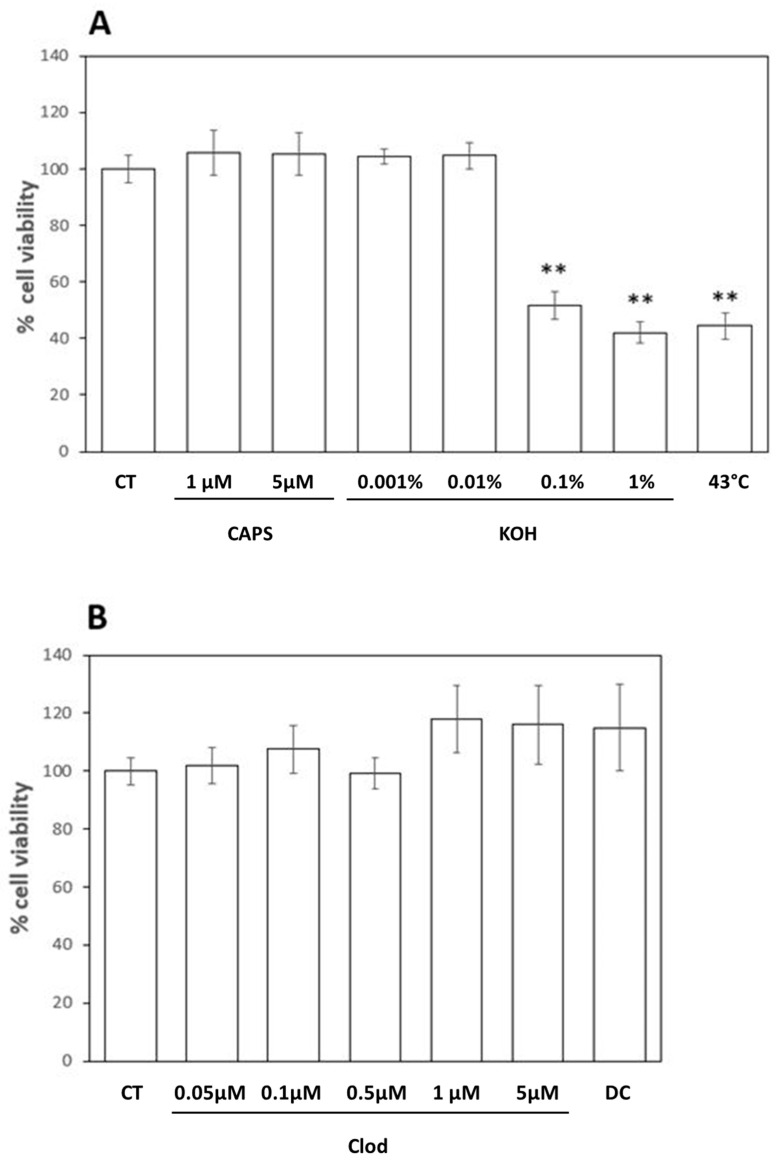
(**A**) MTT toxicity test for various nociceptive stimuli. Results were expressed as percentage of viability compared to control (unstimulated) samples. ** *p* > 0.001; (**B**) MTT toxicity test for Clodronate (Clod) and Dansylcadaverine (DC). Results expressed as percentage of cell viability compared to control samples.

The stimuli used were both chemical and physical in nature; in particular, the keratinocytes were exposed to different concentrations of KOH, to Capsaicin, and to a temperature of 43 °C. An essential element to be able to evaluate the onset and transmission of nociceptive signals through the release of vesicles containing ATP was to identify the correct intensity/concentration of nociceptive stimuli that were not lethal at a cellular level. Toxicity analysis using the MTT test provided the data shown in Figure 1A,B and expressed as a % of cell viability indicated as 100% in the negative control (unstimulated) samples ± standard deviation (S.D.).

As shown in Figure 1A, both Capsaicin concentrations tested (1 and 5 µM) showed no toxicity after 3 h of incubation with human keratinocytes (respectively, 106 ± 8% and 105 ± 8% compared to CT samples), while only two of the concentrations of KOH used (0.001% and 0.01%) did not showed toxicity (respectively, 105 ± 3% and 105 ± 5% compared to CT samples). Unfortunately, the stimulation at 43 °C turned out to be extremely toxic after 3 h of incubation (<50% viability), which is why this stimulus was no longer used in our experimentation.

The aim of our study was mainly to evaluate the effect of Clodronate on the transmission of nociceptive stimuli by keratinocytes, for which we also tested in a precautionary manner the toxicity of various concentrations of Clodronate (Clod 0.05–5 µM) and of an endocytosis inhibitor of this bisphosphonate, Dansylcadaverine (DC, 0.8 µM). As shown in Figure 1B, no concentration of Clodronate was toxic after 3 h of incubation; indeed, the presence of Clodronate seemed to increase cell viability, although not significantly. The same absence of toxicity was observed after exposure to DC.

### 2.2. MV Release Induced by Nociceptive Stimuli

The release of MVs in serum-free culture medium by human keratinocytes, stimulated with Capsaicin or KOH, was evaluated using the “Exon View R100 Imager” instrument. As described in the Materials and Methods section, the supernatant was appropriately diluted 1:2 and added for approximately 16 h to a microchip capable of immobilizing the MVs on its surface to allow their visual counting. According to toxicity test results, Capsaicin (5 µM) and KOH (0.01%) were used for a 3 h stimulation at 37 °C in a 5% CO_2_ atmosphere.

**Figure 2:**
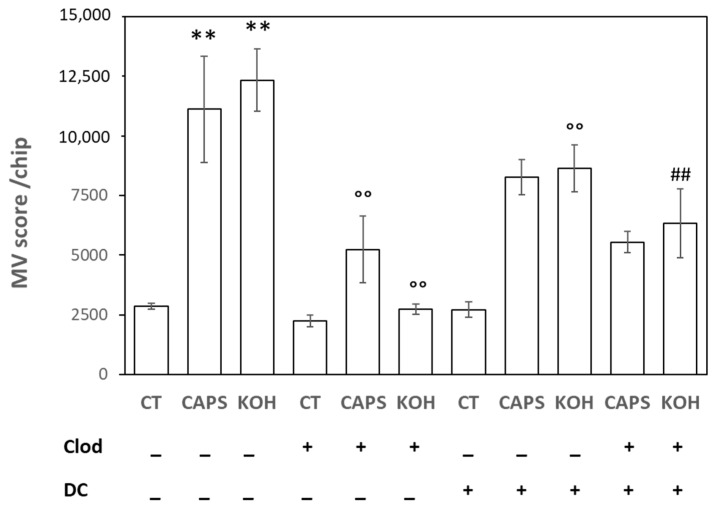
Quantitative analysis of basal (CT)- and nociceptive stimuli (Caps 5 µM, KOH 0.01%)-induced microvesicle release in the presence or absence of Clodronate (Clod, 5 µM) and/or Dansylcadaverine (DC, 0.8 µM). ** *p* < 0.001 compared to unstimulated samples (CT); °° *p* < 0.001 compared to stimulated samples in the absence of Clod; ## *p* < 0.001 compared to stimulated samples in the presence of Clod.

As shown in Figure 2, both nociceptive stimuli were able to increase the release of MVs by 389 ± 78% and 431 ± 46%, respectively, compared to the release observed in the control (unstimulated) samples. This notable increase in the release of MVs was significantly reduced when the cells were pre-incubated for 3 h with 5 µM Clodronate, without any significant effects on the control samples. In fact, the release induced by Capsaicin decreased to 232 ± 62% of the control, while the release induced by KOH was almost abolished (121 ± 10%).

In order to understand the mechanism of action of Clodronate toward the release of MVs induced by nociceptive stimuli, HaCaT cells were pre-incubated for 1 h with 0.8 µM of DC. 

Surprisingly, DC reduced the effect of both Caps and KOH in the absence of Clod without affecting the basal level of MV release (Figure 2). Furthermore, in the presence of Clod, DC was not able to revert the Clod inhibitory effect on Caps-induced MV release, and it partially reverted the one induced on KOH (Figure 2). 

### 2.3. ATP

**Figure 3:**
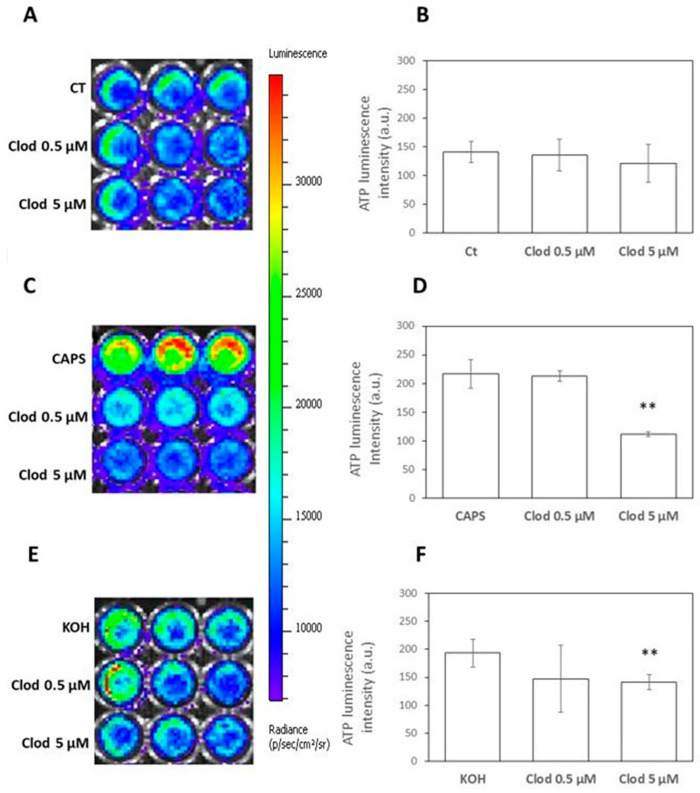
Representative images of ATP luminescence in MVs obtained from HaCaT-conditioned medium in unstimulated samples (CT) (**A**) treated with Caps (5 µM) (**C**) or KOH (0.01%) (**E**) and quantitative analysis of ATP luminescence intensity measured in 3 different experiments in CT (**B**), Caps (**D**), and KOH (**F**) samples. ** *p* < 0.001 compared to unstimulated samples.

Since ATP is one of the nociception mediators, we evaluated its presence in MVs released by human keratinocytes in both basal and stimulated conditions. As indicated in the Materials and Methods section, ATP was measured using a method based on the luciferin–luciferase reaction. This method could not be coupled with the analysis of MVs on chip due to the low intensity of the signal given by luciferin, so the presence of ATP was analyzed after lysis of the MVs using the “IVIS Spectrum In Vivo Imaging System” instrument. In the control samples, the presence of Clod (0.5 and 5 µM) (Figure 3A,B) did not modify the quantity of the luminescence signal derived from the presence of ATP. In the samples stimulated with Capsaicin (Figure 3C,D) and KOH (Figure 3E,F), a notable increase in the signal derived from the presence of ATP was observed as expected. The increased ATP signal was then inhibited in the samples treated with nociceptive stimuli by the presence of Clodronate in a dose-dependent manner. These latest results, in addition to confirming the hypothesis that nociceptive stimuli are able to increase the release of MVs and ATP, have demonstrated that Clodronate is able to inhibit both phenomena, but in a more specific way, the accumulation of ATP within the MVs, confirming the mechanism of action underlying the analgesic activity observed for this non-N-BP. 

## 3. Discussion

In this study, we first demonstrated that human keratinocytes stimulated in vitro by Capsaicin or KOH, two well-known nociceptive stimuli [22], increased MV shedding and ATP release, and then we showed that Clodronate was able to reduce the shedding of ATP-laden MVs.

Capsaicin is an agonist of TRPV1 (Transient Receptor Potential Vanilloid 1), a Ca^2+^-selective member of the family of transient release potential ion channels, which sense heat [23]. TRPV1 is broadly expressed in different human organs (brain, bladder, liver, and kidneys) and in epidermal keratinocytes, polymorphonuclear granulocytes, and macrophages [24]. After Capsaicin activation, TRPV1 mediates Ca^2+^ influx and ATP release [25]. Because topical application of Capsaicin produces burning pain in humans and animals, it has been used to produce standardized experimental pain to test the effectiveness of newly developed analgesic compounds. Importantly, high topical concentrations of Capsaicin (8%, 0.2 M) have long been used as an analgesic agent, inducing ablation of TRPV1+ nerve terminals [26]. KOH is an inorganic base considered a strong skin irritant able to induce ATP release from keratinocytes [22] and Ca^2+^ flux without disrupting the cellular membranes [27]. Both Capsaicin and KOH stimulated the release of MVs from HaCaT cells. Microvesicles (MVs) are irregularly shaped extracellular vesicles with a 100–1000 nm diameter [28], and their biogenesis is induced by an increase in intracellular calcium, which leads to changes in the membrane lipid distribution and the disruption of cytoskeleton integrity [29]. MV secretion has been identified as a novel and universal mode of intercellular communication between cells. MVs can contain various soluble proteins, lipids, DNA, and RNA, as well as organelles such as mitochondria [30] and their involvement in both physiological (immune response [17], wound healing [31], aging [32], etc.) and pathological (cancer [33] and neurodegenerative disorders [34]) processes has been largely proven. The production of microvesicles has currently never been associated with the transmission of pain stimuli, even if recent findings indicated that MVs containing purinergic enzymes and molecules are involved in immune modulation [18] and ATP-loaded MVs are released in the tumoral microenvironment by nutrient deprivation [18]. In fact, MVs are released spontaneously into the extracellular medium from the cells, but their release can be strongly increased by modifying cultivation conditions (starvation, hypoxia) and/or applying different chemical (H_2_O_2_, Ca_2_) or physical (shear stress, ultrasounds) stress stimuli [16]. In our experimental model, both Capsaicin and KOH can be considered stress stimuli that are able to induce both MV shedding and ATP release through a probable increase in intracellular Ca^2+^ concentration. We also observed that cell pretreatment with Clod decreased the ATP-loaded MV shedding induced by Capsaicin and KOH in a dose-dependent fashion. Clod is a first-generation bisphosphonate, analgesic in nature, acting as a selective and potent inhibitor for vesicular nucleotide transporter (VNUT) responsible for vesicular storage of ATP [35]. Clod reduces chronic neuropathic and inflammatory pain, inhibiting vesicular ATP release from neurons and blocking purinergic chemical transmission [35]. It is noteworthy that keratinocytes also express VNUT, which is involved in *Candida* nociception [8]. MVs released in the intercellular space can be taken up by surrounding cells by endocytosis or phagocytosis and release their content intracellularly, modulating cellular functions, even if this uptake seems to be a low-yield process [36]. Therefore, ATP or adenosine contained in MVs may be released into the cytoplasm of the target cell, such as peripheral neurons, but as ATP intracellular concentration is higher than in the extracellular space, MV-derived ATP will not modify intracellular concentration. Otherwise, MVs could release adenine nucleotides in proximity to target cells interacting with purinergic receptors eventually present on the plasmatic membrane. All these elements allow us to hypothesize that the release of MVs loaded with ATP could be an alternative method of transmitting nociceptive information from the first cells that are stimulated or damaged, in this case, the keratinocytes of the skin or of a mucosa, up to the peripheral nerve endings that will lead to the creation of pain perception at the central nervous system level. Furthermore, the ability of Clodronate to completely inhibit both the formation of MVs and the accumulation of ATP at a much lower concentration (5 µM) than that normally used in in vitro studies (30–1000 µM) [37] suggests that this drug could become an interesting topical analgesic. Unfortunately, to reach these results, it is mandatory to create the conditions for a better and rapid uptake of Clod by the cells of the skin or mucous. In fact, Clod is uptake through slow endocytosis [37] followed by vesicular acidification [38]; therefore, in order to enhance its efficacy, it is often encapsulated in liposomes [36]. In our study, we tried to inhibit Clod uptake using Dansylcadaverine (DC) [38], an endocytosis inhibitor that showed both an ability to reduce MV shedding in Caps- and KOH-treated samples in the absence of Clod, probably due to its ability to interfere with the plasma membrane and a reduced ability to revert the Clod inhibition of MV-induced release, maybe due to a low DC concentration.

This preliminary work has some limitations. First, the use of a cell line of spontaneously immortalized human keratinocytes could express levels of VNUT significantly different from those found in primary keratinocytes and, therefore, could behave differently if stimulated with Clodronate. Furthermore, precisely because this is an exploratory study, only one Dansylcadaverine concentration was used to inhibit Clodronate uptake, which does not always seem to have acted with the expected efficacy. Despite this, a relationship appears to emerge from this study between VNUT activity and the production of MVs, a relationship that should be analyzed using cellular models of overexpression or inhibition of the expression of this transporter.

## 4. Materials and Methods

### 4.1. Cell Culture

The human spontaneously immortalized keratinocyte cell line HaCaT was used for all experiments [39]. The cells were obtained from Cell Lines Service GmbH (Eppelheim, Germany™) and cultured in 25 cm^2^ plastic flasks. Dulbecco’s modified Eagle medium (DMEM) with high glucose levels, supplemented with 10% heat-inactivated fetal bovine serum (FBS) and 1% penicillin–streptomycin (all from Immunological Sciences, Rome, Italy), was used as the culture medium. The cells were incubated in a humidified incubator at 37 °C with 5% CO_2_. Phosphate-buffered saline (PBS 1×, pH = 7.2) was used for sterilization and experimental procedures. For toxicity, MV release, and ATP measure experiments, HaCaT cells were detached using trypsin (0.25% trypsin in PBS containing 0.05% EDTA), resuspended, and seeded in 24-well or 96-well plates at a density of 1 × 10^5^ cells per well in complete DMEM. The cells were allowed to adhere and grow until they reached 100% confluence. Once confluent, the cells were washed twice with PBS 1× and then cultivated in serum-free DMEM for 24 h at 37 °C with 5% CO_2_.

### 4.2. MTT Assay

HaCaT cells were stimulated using three different agents capable of inducing nociception in vivo. In a series of preliminary experiments, the toxicity of the various nociceptive stimuli was evaluated in order to identify the experimental conditions that did not induce cell mortality significantly higher than that observed in the control (unstimulated) samples. The three nociceptive agents were Capsaicin (Caps, 1–5 µM), KOH (0.001–0.1%), and a temperature of 43 °C [21,22]. During this first series of experiments, we also evaluated the toxicity of various concentrations of Clodronate (Clod, 0.05–5 µM), chosen based on Clodronate IC50 for VNUT [35] and previous in vitro studies [36], and Dansylcadaverine (DC, 0.8 µM), an inhibitor of Clod uptake [37,40]. Cell viability was evaluated by MTT assay, an experimental approach based on tetrazolium salts reduction [41]. Confluent HaCaT in 96-well cell culture plates were incubated in the different conditions of interest for 3 h in serum-free DMEM. At the end of the incubation time, cells were incubated in cell culture medium without phenol red (Euroclone, Milan, Italy) containing 0.5 mg/mL MTT (Thermo Fisher Scientific, Waltham, MA, USA) for 4 h at 37 °C to allow formazan salts precipitation. The resulting insoluble purple precipitate was then dissolved in DMSO (Carlo Erba Reagents, Cornaredo, Italy), and the absorbance was read at 570 nm using a microplate reader (Victor X4, Perkin-Elmer, Waltham, MA, USA). Cell viability was expressed as % of absorbance in untreated samples.

### 4.3. MV Analysis

MVs released in HaCaT cells conditioned medium were analyzed using ExoView Tetraspanin chips (NanoView Biosciences, Boston, MA, USA) arrayed with antibodies against the CD81, CD63, and CD9 proteins. Mouse IgG1 was used as a negative control, and chips were read using an ExoView R100 reader (NanoView Biosciences, Boston, MA, USA). Confluent HaCaT cells seeded in 24-well plates were untreated (control samples, Ct) or treated with Caps 5 µM or KOH 0.01% for 3 h. The 43 °C stimulation was not used for MV release because of its high toxicity. In some experiments, cells were pre-incubated for 3 h with 5 µM Clod to evaluate the ability of the non-N-BP to inhibit MV release. In some additional experiments, cells were treated for 1 h with 0.8 µM DC before Clod preincubation to evaluate the effect of this uptake inhibitor on the non-N-BP activity. At the end of the different incubation periods, the cell-conditioned medium was collected and stored at −20 °C for no more than three days before analysis. Chips were handled according to manufacturer indication. Briefly, the conditioned medium was diluted 1:2 in the incubation solution at room temperature, and 50 μL of diluted sample was applied to each chip. Plates were sealed and incubated for 16 h at room temperature in the dark. Chips were then washed 3 times with solution A. After each wash, the plate was shaken at 500 rpm (LSE Digital Microplate Shaker, Corning, Glendale, AZ, USA) for 3 min. Following the final wash, 250 μL of kit-provided blocking solution was added to each well. The plates were incubated for 1 h at room temperature in the dark. Wells containing chips were then washed 5 times, the first wash in solution A, the next 3 washes in solution B, and a final wash in 0.1 μm filtered distilled water. Chips were carefully removed and placed in Petri dishes (10 cm diameter) containing 1 μm filtered distilled water. Chips were washed, dried, and imaged using the ExoView R100 reader using ExoViewer 3.14 software. The data were exported using ExoView Analyser 3.0 with fluorescence gating based on control mouse IgG capture. Sizing thresholds were set from a diameter of 50 to 200 nm. The resulting microvesicles’ total number information for each individual condition was exported to Excel for Microsoft 364 software (version 2406) for statistical analyses. 

### 4.4. ATP Measure

The ATP test is a method used to quantify the number of viable cells in culture, evaluating the amount of ATP present, which represents metabolically active cells. The “CellTiter-Glo^®^ 2.0 Assay” kit (Promega Italia Srl, Milan, Italy) was used to evaluate the ATP present in the MVs released in the cell-conditioned medium. Bioluminescent ATP assays exploit the firefly luciferase enzyme reaction, which utilizes ATP to produce light. MVs are lysed to release ATP for measurement, and reagents containing firefly luciferase and its substrate are added to drive a two-step reaction. In the first step, luciferin is activated by ATP, forming luciferyl-adenylate and pyrophosphate. In the second step, luciferyl-adenylate reacts with oxygen to create oxyluciferin in an excited state along with CO_2_. The excited oxyluciferin then transitions to a ground state, emitting green to yellow luminescent light (550–570 nm). The luminometer detects the intensity of this luminescent signal. When ATP is the limiting factor in the luciferase reaction, the luminescence is directly proportional to the ATP concentration. Thus, a stronger luminescent signal corresponds to higher ATP levels. The supernatant aliquots of untreated (CT) or treated with Caps or KOH samples in the absence or presence of 0.5 and 5 µM Clod were thawed at room temperature, as well as the reagent for ATP measurement. The samples were centrifuged at 2500× *g* for 15 min, then 30 µL of each sample was added in triplicate to a 96-well plate. An equal volume of adenosine triphosphate (ATP) reagent was added to wells, and plates were placed on the rocking shaker for 10 min (30 rpm) in the dark. Finally, the bioluminescence of the samples was analyzed using the IVIS Spectrum In Vivo Imaging System instrument (Revvit, Waltham, MA, USA).

### 4.5. Statistical Analysis

Each experiment was conducted in triplicate to ensure statistical significance. Statistical analysis was performed using one-way ANOVA followed by Bonferroni’s post hoc tests. The Prism 4.0 statistical software (GraphPad Software Inc., Boston, MA, USA) was used for the analysis. Probability values of *p* < 0.05 were considered statistically significant.

## 5. Conclusions

In conclusion, for the first time, two nociceptive stimuli applied in vitro to human keratinocytes were able to induce the release of ATP-containing MVs, a possible pain transducer, and Clod, a first-generation bisphosphonate with important analgesic activities, was able to inhibit this MV release. Even if this new ATP release mechanism needs a more accurate investigation, mainly to confirm our results also in different cellular models and to understand the relationship between VNUT and MV shedding mechanism, our results shed new light on a possible use of the “old” drug Clodronate as a new topical analgesic to be used to prevent pain in small skin or mucosa areas or to reduce pain associated with small tissue damage. To achieve these objectives, not only will preclinical studies be necessary using animal models in which to evaluate the real inhibition of pain, but also a different formulation of Clodronate, which so far has been used in forms for intravenous, oral, or intramuscular administration.

## Data Availability

The data presented in this study are available upon request from the corresponding author as this paper is the first one of a three-paper project for a patent deposition.
